# Supplementary material for: Thawing Yedoma permafrost is a neglected nitrous oxide source
Source: Nat Commun. 2021 Dec 7;12:7107. doi: 10.1038/s41467-021-27386-2 (PMC8651752; doi:10.1038/s41467-021-27386-2)
Supplement: Supplementary file 2 — Supplementary Information [file 41467_2021_27386_MOESM2_ESM.pdf]

Supplementary Material

For article ‘Thawing Yedoma permafrost is a neglected nitrous oxide source’

By Marushchak M.E., Kerttula J., Diáková K., Faguet A., Gil J., Grosse G., Knoblauch C., Lashchinskiy N., Martikainen P.J., Morgenstern A., Nykamb M., Ronkainen J.G., Siljanen H.M.P., van Delden L., Voigt C., Zimov N., Zimov S. & Biasi C.

Supplementary Figures

Supplementary Figure 1. Studied surfaces at the Kurungnakh exposure

Supplementary Figure 2. Studied surfaces at the Duvanny Yar exposure

Supplementary Figure 3. Shoreline retreat of the Kurungnakh Yedoma exposure as a result of permafrost thaw

Supplementary Figure 4. Vertical subsidence due to thaw and erosion in the Kurungnakh Yedoma exposure

Supplementary Figure 5. Estimation of nitrous oxide (N_2O) emission from Yedoma under dead plants at the Duvanny Yar exposure

Supplementary Figure 6. Scatterplots of nitrous oxide (N_2O) flux against carbon dioxide (CO_2) and methane (CH_4) fluxes and soil physico-chemical characteristics

Supplementary Figure 7. Results of quantitative PCR at the Kurungakh exposure

Supplementary Figure 8. The relative abundance of genes involved in key processes of nitrogen cycle: nitrogen fixation (*nifH*), nitrification (bacterial and archaeal *amoA*), nitrate reduction (*narG, napA*), denitrification (*nir* (*nirK+nirS*)*, norB, nosZ*), DNRA (*nrfA*) & anammox (*hdhA*)

Supplementary Figure 9. Relative abundance of N cycling genes at the Kurungnakh exposure from all functional gene sequences captured with the targeted metagenomics tool.

Supplementary Figure 10. Effect of drying (25% reduction in water content), C addition (glucose) and C+N addition (glucose+NO_3^-) on N_2O production under different headspace conditions.

Supplementary Figure 11. Disturbed Yedoma sites with potential for high N_2O emissions across the circum-arctic Yedoma region.

Supplementary Tables

Supplementary Table 1. Field N_2O fluxes and soil parameters at the Kurungnakh and Duvanny Yar Yedoma exposures

Supplementary Table 2. Soil physical and chemical characteristics at the Kurungnakh and Duvanny Yar Yedoma exposures

Supplementary Table 3. Nitrogen transformation rates at the Kurungnakh Yedoma exposure

Supplementary Table 4. Nitrous oxide production rates at the Kurungnakh Yedoma exposure under different headspace conditions

Supplementary Table 5. Primer pairs used in this study and corresponding amplification protocols

Supplementary Notes

Supplementary Note 1. Extended methods.

Supplementary Figures

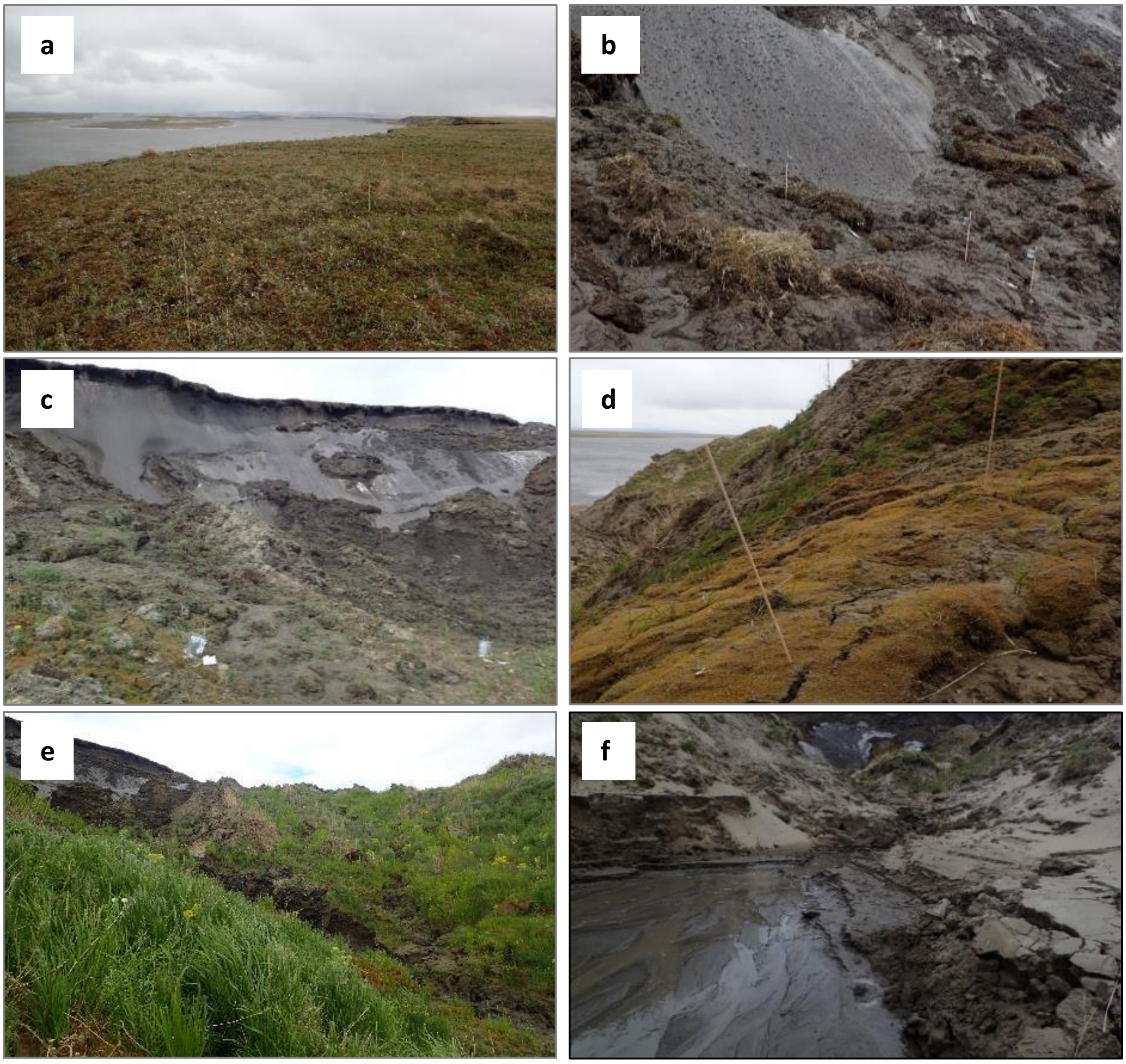

Supplementary Figure 1. Studied surfaces at the Kurungnakh exposure. a, Vegetated Holocene cover deposits overlying undisturbed Yedoma on the top of the exposure. **b**, Bare freshly thawed Yedoma. **c**, Bare earlier thawed Yedoma. **d**, Disturbed Yedoma revegetated with mosses. **e**, Disturbed Yedoma revegetated with grasses. **f**, Bare sand on river shore receiving Yedoma melt waters.

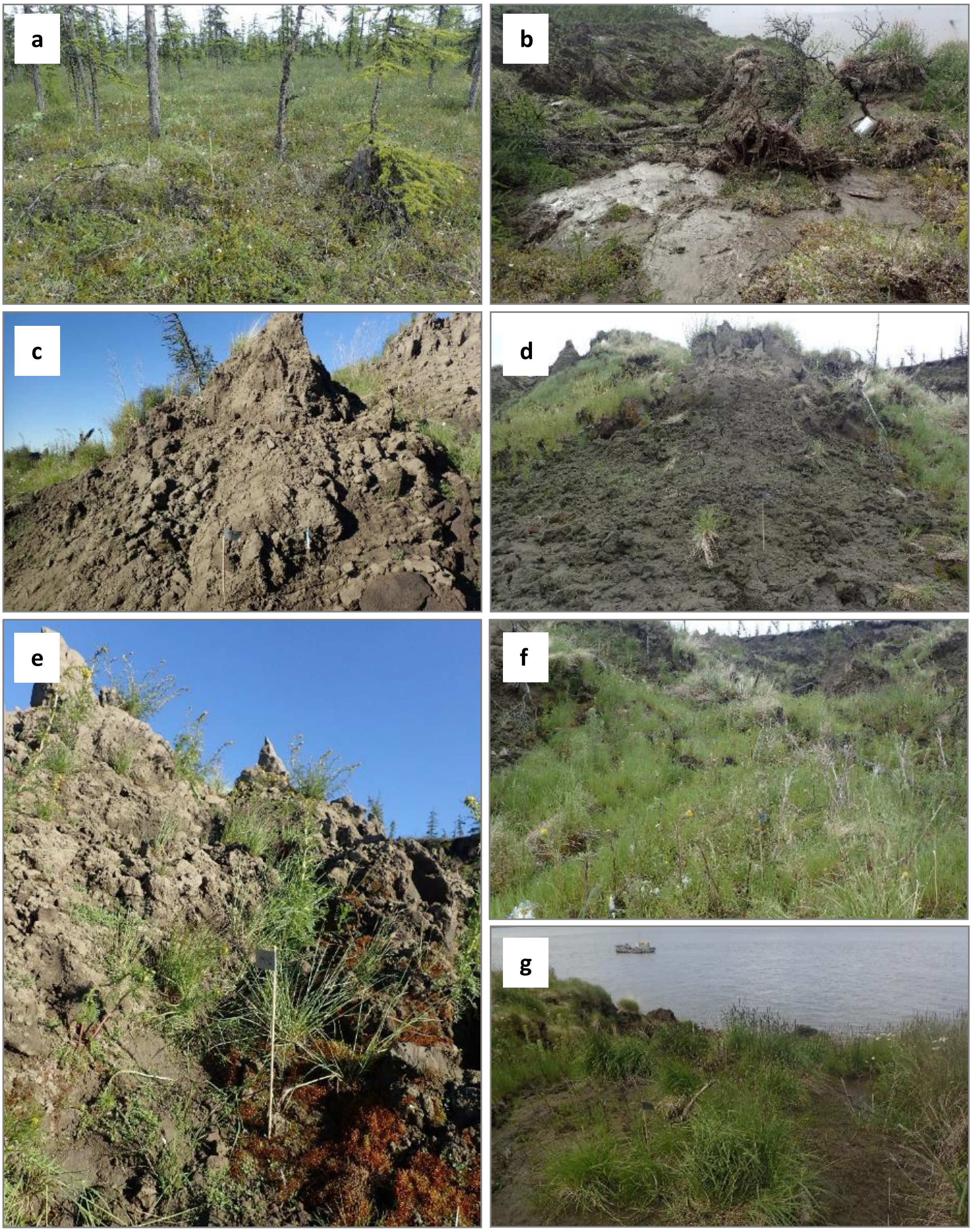

Supplementary Figure 2. Studied surfaces at the Duvanny Yar exposure. **a**, Vegetated Holocene cover on the top of the exposure. **b**, Bare freshly thawed Yedoma. **c&d**, Bare earlier thawed Yedoma. **e**, Disturbed Yedoma revegetated with mosses. **f&g**, Disturbed Yedoma revegetated with grasses.

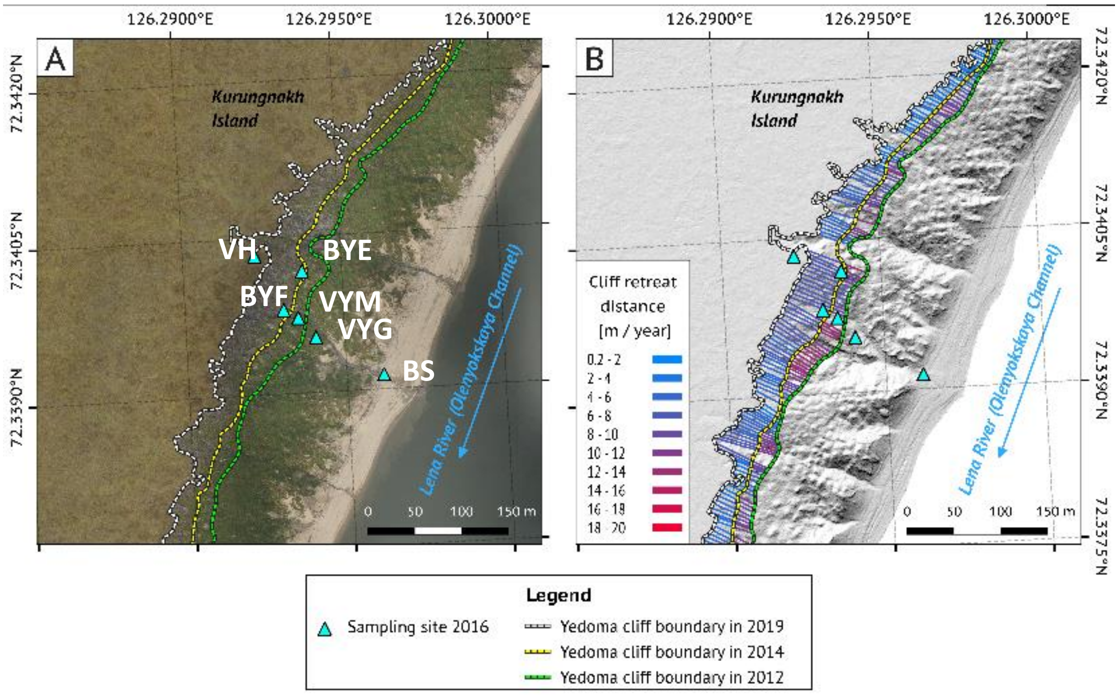

Supplementary Figure 3. Shoreline retreat of the Kurungnakh Yedoma exposure as a result of permafrost thaw. a, Unmanned aerial vehicle (UAV) imagery of the study site (Aug. 2019) on Kurungnakh Island. **b,** Rate of Yedoma cliff boundary retreat calculated from Arctic DEM (Digital Elevation Model; March 2012 and 2014) and UAV data (August 2019). The sampling sites are indicated with abbreviations in **a**: VH – Vegetated Holocene cover, BYF – Bare freshly thawed Yedoma, BYE – Bare earlier thawed Yedoma, VYM –Yedoma with revegetated mosses, VYG –Yedoma revegetated with grasses, and BS – Bare sand.

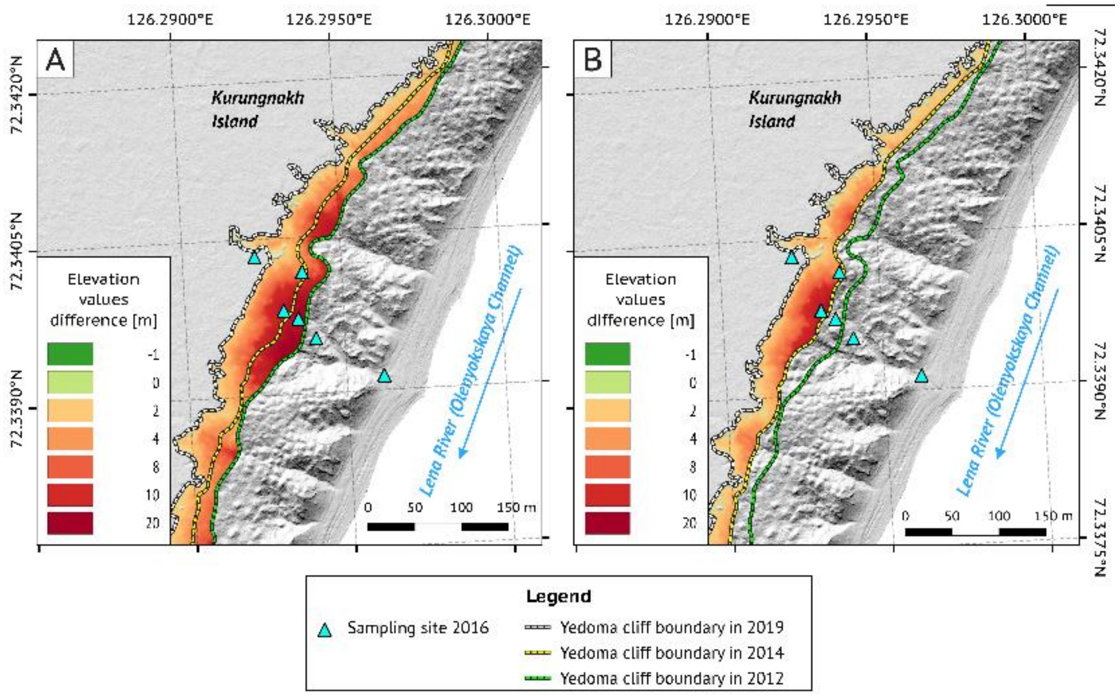

Supplementary Figure 4. Vertical subsidence due to thaw and erosion in the Kurungnakh Yedoma exposure. a, Arctic DEM (Digital Elevation Model), March 2012 and UAV (Unmanned Aerial Vehicle) data, August 2019. **b,** Arctic DEM, March 2014 and UAV data, August 2019. Positive values indicate terrain subsidence.

a **b**

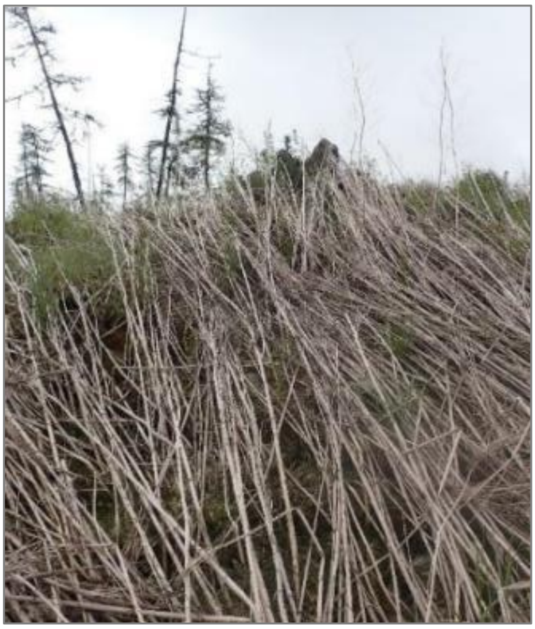

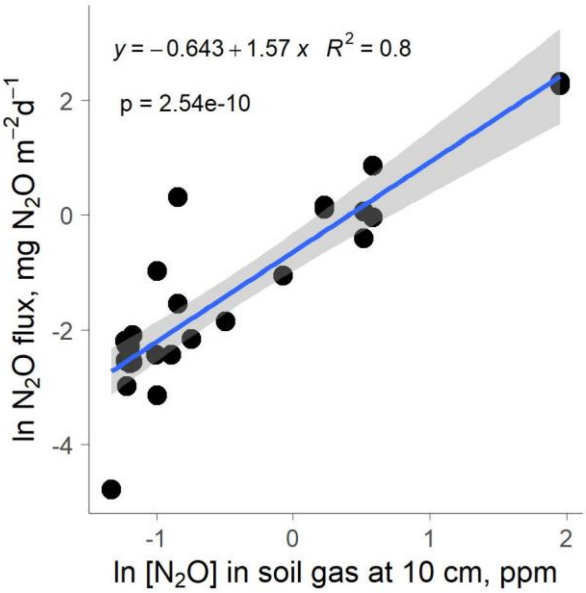

Supplementary Figure 5. Estimation of nitrous oxide (N_2O) emission from Yedoma under dead plants at the Duvanny Yar exposure. a, Picture of the thawed Yedoma surface with high amount of dead biomass of *Descurainia sophioides.* **b**, Dependence of N_2O flux in revegetated Yedoma surfaces on N_2O concentration ([N_2O]) in soil pore gas at 10 cm. The regression function was used for estimating N_2O flux from dead plant sites. Note the logarithmic scale on both axes. The shaded area shows the 95% confidence interval of the regression line.

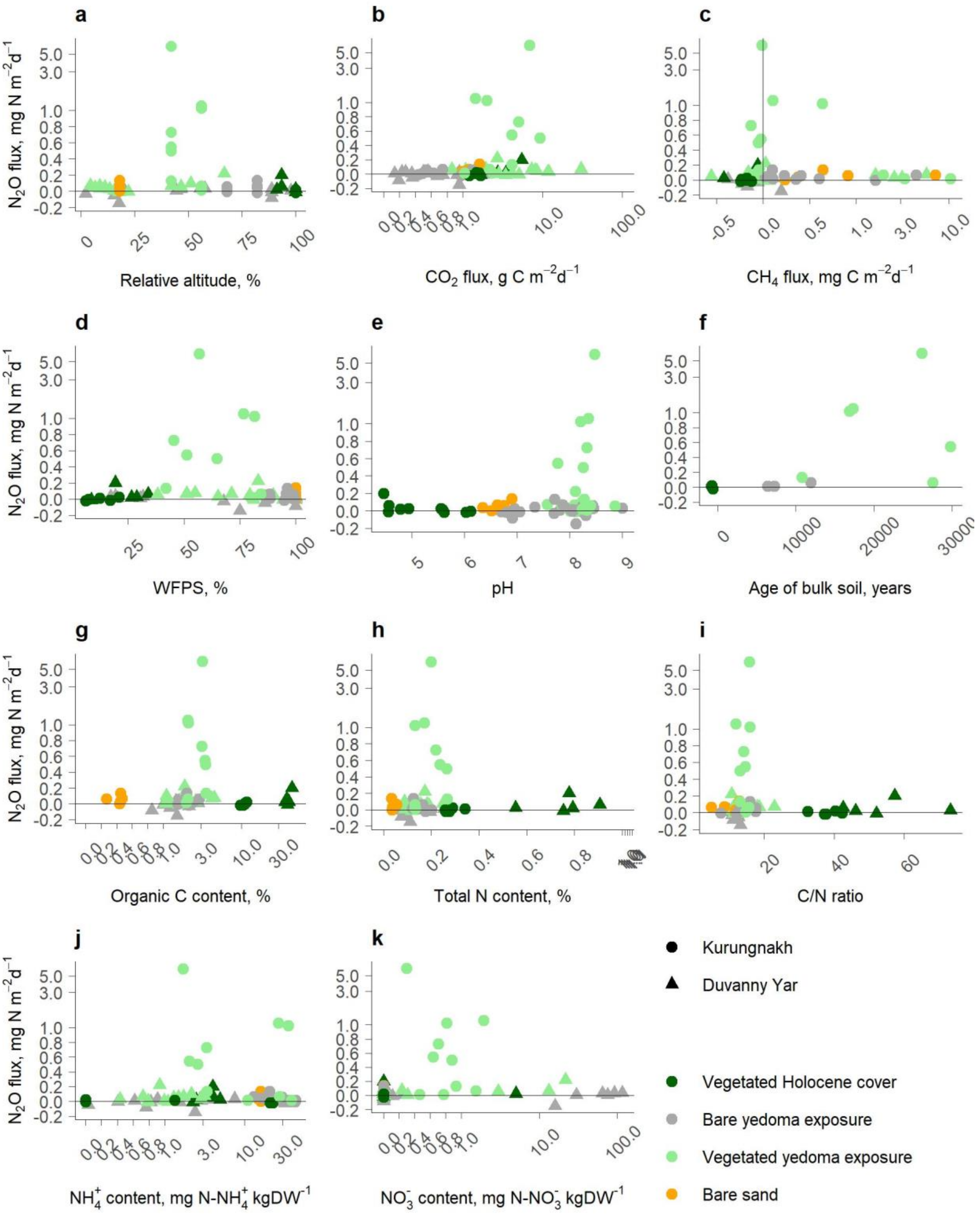

Supplementary Figure 6. Scatterplots of nitrous oxide (N_2O) flux against carbon dioxide (CO_2) and methane (CH_4) fluxes and soil physico-chemical characteristics. a, Relative altitude on the slope. **b**, Carbon dioxide flux measured as ecosystem respiration. **c**, Methane flux. **d**, Water filled pore space (WFPS). **e**, pH. **f**, Radiocarbon age of bulk soil. **g**, Total carbon (C) content. **h**, Total nitrogen (N) content. **i**, Carbon to nitrogen (C/N) ratio. **j**, Ammonium (NH_4^+) content. **k**, Nitrate (NO_3^-) content. Circles indicate data from Kurungnakh, triangles indicate data from Duvanny Yar. Different colors indicate different surface types, defined by the legend.

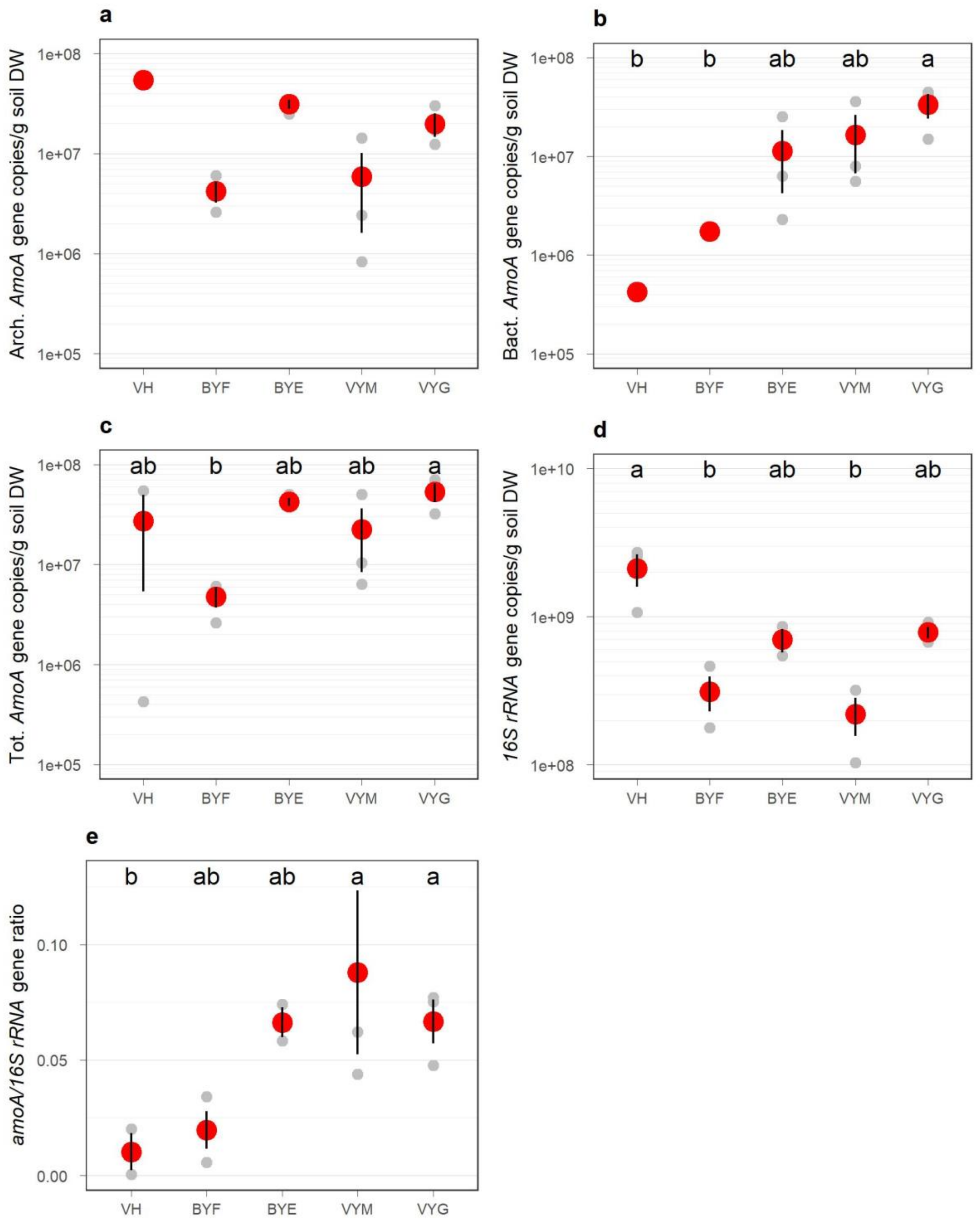

Supplementary Figure 7. Results of quantitative PCR at the Kurungakh exposure. a, Gene copy number of archaeal *amoA*. **b**, Gene copy number of bacterial *amoA.* **c**, Gene copy number of total *amoA* (archaeal + bacterial). **d**, Gene copy number of *16S rRNA*. **e**, Gene ratio of *amoA/16S rRNA*. The studied surfaces are arranged according to the distance from the Yedoma cliff border, with intact Holocene cover on the left and earliest thawed revegetated Yedoma on the right. Small grey symbols indicate values for individual samples, large red symbols indicate means, and error bars indicate standard error of mean (n = 3 biologically independent samples). Lower case letters indicate significant differences between studied soils (Kruskal-Wallis test with pairwise comparisons with Dunn's test; $p < 0.05$). DW = dry weight; VH – Vegetated Holocene cover, BYF – Bare freshly thawed Yedoma, BYE – Bare earlier thawed Yedoma, VYM –Yedoma revegetated with mosses, and VYG – Yedoma revegetated with grasses.

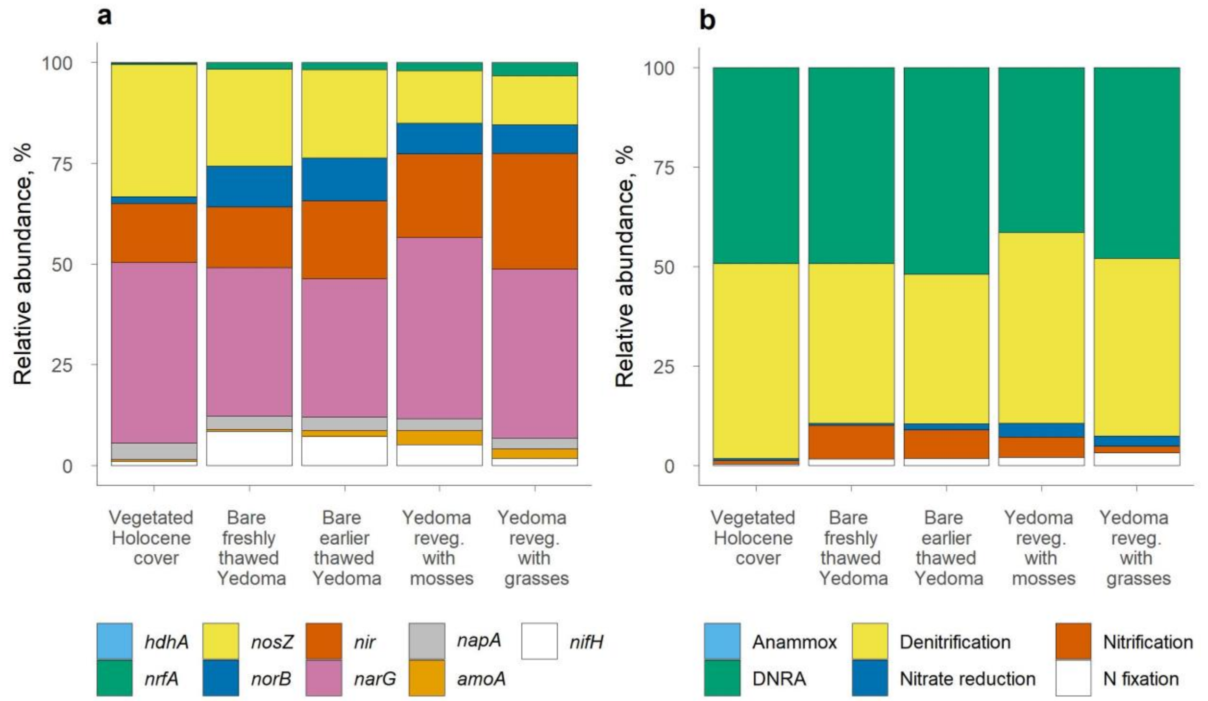

Supplementary Figure 8. The relative abundance of genes involved in key processes of nitrogen cycle: nitrogen fixation (*nifH*), nitrification (bacterial and archaeal *amoA*), nitrate reduction (*narG, napA*), denitrification (*nir (nirK+nirS), norB, nosZ*), dissimilatory nitrate reduction to ammonium (DNRA; *nrfA*) & anammox (*hdhA*). a, Relative abundances separately for each gene. **b**, Relative abundances grouped according to metabolic pathway. The studied surfaces are arranged according to the distance from the Yedoma cliff border, with intact Holocene cover on the left and earliest thawed revegetated Yedoma on the right. n = 3 biologically independent samples.

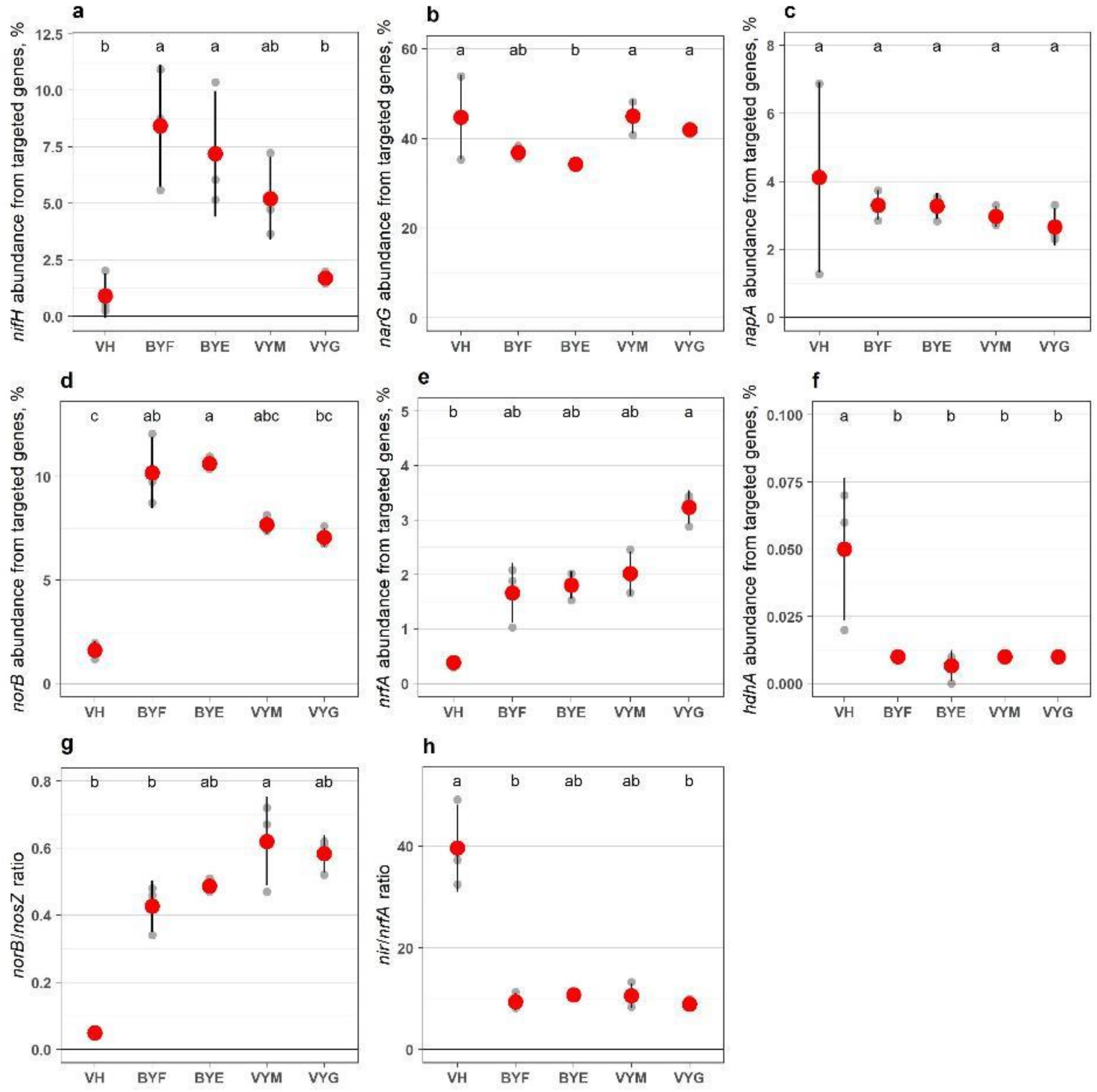

Supplementary Figure 9. Relative abundance of N cycling genes at the Kurungnakh exposure from all functional gene sequences captured with the targeted metagenomics tool. a*,* Relative abundance of *nifH* gene. **b**, Relative abundance of *narG* gene. **c**, Relative abundance of *napA* gene. **d**, Relative abundance of *norB* gene. **e**, Relative abundance of *nrfA* gene. **f**, Relative abundance of *hdhA* gene. **g**, Ratio of norB/*nosZ* genes. **h**, Relative abundance of *nir/nrfA* genes. The studied surfaces are arranged according to the distance from the Yedoma cliff border, with intact Holocene cover on the top of the Yedoma exposure on the left and earliest thawed revegetated Yedoma on the right side. Small grey symbols indicate values for individual samples, large red symbols indicate means, and error bars indicate standard error of mean (n = 3 biologically independent samples). Lower case letters indicate significant differences between studied soils (Kruskal-Wallis test with pairwise comparisons with Dunn's test; unadjusted $p < 0.05$). The relative abundance of *amoA* (including both archaeal and bacterial), *nir* (including *nirK* and *nirS*) and *nosZ* genes, as well as ratio of (*nirK*+*nirS*)/*nosZ* genes are shown in Fig. 4 of the main manuscript. VH – Vegetated Holocene cover, BYF – Bare freshly thawed Yedoma, BYE – Bare earlier thawed Yedoma, VYM –Yedoma revegetated with mosses, and VYG – Yedoma revegetated with grasses.

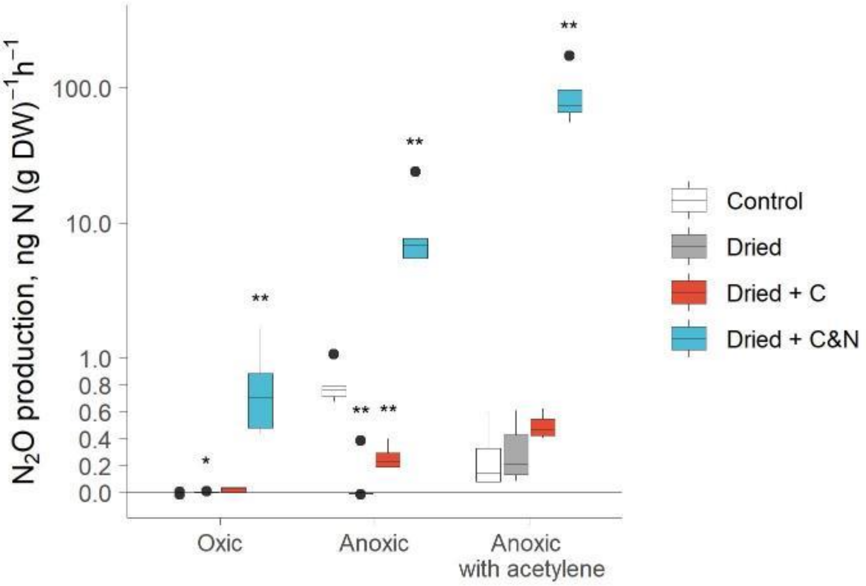

Supplementary Figure 10. Effect of drying (25% reduction in water content), carbon (C) addition (as glucose) and C + nitrogen (N) addition (glucose + nitrate (NO_3^-)) on nitrous oxide (N_2O) production under different headspace conditions. Acetylene was used to inhibit the last step of denitrification (N_2O reduction to N_2), allowing estimation of the total denitrification rate. Box plots show lower and upper quartiles, median (thick black line), smallest and largest values without outliers (thin black line) and outliers (circles); *n* = 5 biologically independent samples. Stars indicate significant differences from control of the same headspace treatment (two-sided Wilcoxon signed-rank test; * unadjusted $p < 0.05$, ** unadjusted $p < 0.01$). Note the logarithmic scale on y-axes.

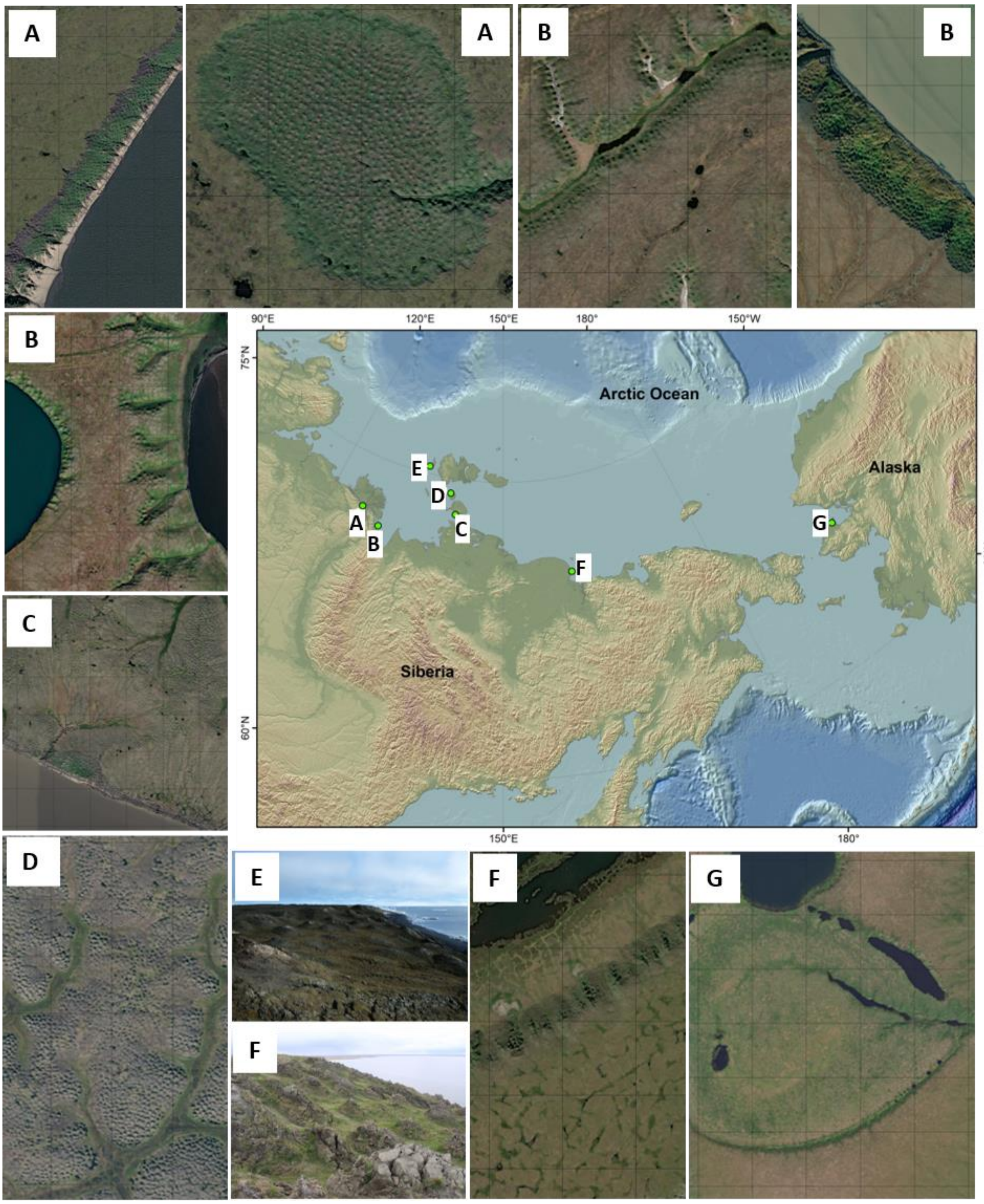

Supplementary Figure 11. Disturbed Yedoma sites with potential for high N_2O emissions across the circum-arctic Yedoma region. The map shows the location of the sites: **A** – Kurungnakh Island, Lena Delta; **B** – Bykovsky Peninsula; **C** – Big Lyakhov Island; **D** – Small Lyakhov Island; **E** – Belkovsky Island; **F** – Mys Chukochy; **G** – Northern Seward Peninsula. The aerial and fied images have been marked with respective site codes. Field photos **E** and **F** by G. Grosse;.

Supplementary Tables

Supplementary Table 1. Field nitrous oxide (N_2O), methane (CH_4) and carbon dioxide (CO_2, ecosystem respiration) fluxes and soil parameters at the Kurungnakh and Duvanny Yar Yedoma exposures. Lower case letters indicate statistically significant differences between surfaces (N_2O flux, CH_4 flux, CO_2 flux, VWC and Soil T: Kruskal-Wallis test with pairwise comparisons with Dunn's test; WFPS: ANOVA with pairwise tests with Games-Howell post hoc test; $p < 0.05$), tested separately within each study site. For bare earlier thawed Yedoma and revegetated Yedoma with grasses in Duvanny Yar $n = 10$, for all other surfaces $n = 5$. VWC – volumetric water content; WFPS – water filled pore space; Soil T – soil temperature; SD – standard deviation. For N_2O, CH_4 and CO_2 fluxes, positive values indicate emissions, and negative values indicate uptake.

	**N_2O flux** **μg N m^{-2} d^{-1}**				**CH_4 flux** **mg C m^{-2} d^{-1}**				**CO_2 flux** **mg C m^{-2} d^{-1}**				**VWC** **m^3 m^{-3}**			**WFPS** **%**			**Soil temp., 10 cm** **°C**		
	**Median**	**Min**	**Max**	**Sig.**	**Median**	**Min**	**Max**	**Sig.**	**Median**	**Min**	**Max**	**Sig.**	**Mean**	**SD**	**Sig.**	**Mean**	**SD**	**Sig.**	**Mean**	**SD**	**Sig.**
**KURUNGNAKH**																					
**Vegetated Holocene cover**	–4	–19	22	**b**	–0.18	–0.24	–0.13	**c**	1584	1192	1652	**ab**	0.12	0.06	**b**	13	6	**d**	1.3	0.9	**c**
**Bare freshly thawed Yedoma**	16	–3	61	**b**	0.41	0.11	1.62	**ab**	626	442	1292	**b**	0.51	0.06	**a**	94	5	**a**	6.4	1.2	**a**
**Bare earlier thawed Yedoma**	58	–18	135	**ab**	0.10	–0.13	4.45	**abc**	740	540	971	**b**	0.46	0.03	**a**	94	5	**a**	4.4	0.5	**abc**
**Yedoma revegetated with mosses**	64	16	1147	**ab**	1.95	0.10	10.34	**a**	1985	1041	2333	**ab**	0.40	0.01	**ab**	81	3	**b**	5.9	0.3	**a**
**Yedoma revegetated with grasses**	548	133	6286	**a**	–0.05	–0.13	–0.01	**bc**	5019	4069	9228	**a**	0.31	0.05	**b**	52	9	**c**	5.2	0.6	**a**
**Bare sand**	62	0	136	**ab**	0.64	0.24	7.10	**a**	1150	988	1607	**b**	0.50	0.02	**a**	100	0	**a**	3.3	0.4	**bc**
**DUVANNY YAR**																					
**Vegetated Holocene cover**	26	–12	200	**ab**	–0.15	–0.42	0.07	**a**	3479	1667	5487	**a**	0.23	0.10	**b**	23	10	**c**	3.9	0.6	**b**
**Bare freshly thawed Yedoma**	–9	–84	27	**b**	–0.11	–0.38	0.10	**a**	234	139	923	**b**	0.44	0.02	**a**	100	0	**a**	9.2	1.0	**a**
**Bare earlier thawed Yedoma**	9	–147	34	**b**	–0.04	–0.23	0.19	**a**	549	279	1839	**b**	0.29	0.16	**b**	55	34	**bc**	9.7	3.0	**a**
**Yedoma reveg. with mosses**	73	56	222	**a**	–0.16	–0.18	0.03	**a**	1574	861	2693	**ab**	0.36	0.07	**b**	69	24	**abc**	10.0	0.8	**a**
**Yedoma reveg. with grasses**	30	–11	70	**ab**	0.08	–0.56	5.73	**a**	7639	2822	30374	**a**	0.46	0.04	**a**	79	18	**b**	9.6	3.2	**a**

Supplementary Table 2. Soil physical and chemical characteristics at the Kurungnakh and Duvanny Yar Yedoma exposures. Samples were taken from the depth of 0–10 cm. Lower case letters indicate statistically significant differences between surfaces (bulk density, soil organic carbon (SOC) content, carbon-13 isotopic signature of soil organic carbon ($\delta^{13}C_{SOC}$), Radiocarbon age, Total nitrogen (N) content, carbon to nitrogen (C/N) ratio, 15-nitrogen isotopic signature of bulk soil ($\delta^{15}N_{bulk}$), ammonium (NH_4^+) content, nitrate (NO_3^-) content, pH: Kruskal-Wallis test with pairwise comparisons with Dunn's test; soil organic matter (SOM), Total C content: ANOVA with pairwise tests with Games-Howell post hoc test; $p < 0.05$), tested separately within each study site. For bare earlier thawed Yedoma and revegetated Yedoma with grasses in Duvanny Yar n = 10, for all other surfaces n = 5. DW – dry weight; SD – Standard deviation; n.d. – not determined.

**Supplementary Table S2. – 1/2**	**Bulk density g cm^{-3}**			**SOM content %**			**Total C content %**			**SOC content %**			**$\delta^{13}C_{SOC}$ ‰**			**Radiocarbon age Years BP**		
	**Mean**	**SD**	**Sig.**	**Mean**	**SD**	**Sig.**	**Mean**	**SD**	**Sig.**	**Mean**	**SD**	**Sig.**	**Mean**	**SD**	**Sig.**	**Mean**	**SD**	**Sig.**
**KURUNGNAKH**																		
**Vegetated Holocene cover**	0.09	0.01	**c**	26.96	2.55	**a**	11.93	1.54	**a**	10.77	0.73	**a**	-28.0	0.1	**c**	-688	102	b
**Bare freshly thawed Yedoma**	1.22	0.03	**bc**	5.09	1.17	**c**	2.24	0.56	**c**	2.07	0.57	**bc**	-26.2	0.9	**bc**	8 550	2 974	ab
**Bare earlier thawed Yedoma**	1.32	0.03	**ab**	5.09	0.21	**c**	2.06	0.09	**c**	1.96	0.17	**bc**	-24.1	0.1	**a**	n.d.	n.d.	
**Yedoma revegetated with mosses**	1.30	0.09	**ab**	5.41	0.21	**c**	2.03	0.04	**c**	2.02	0.05	**bc**	-24.9	0.1	**ab**	20 597	6 046	ab
**Yedoma revegetated with grasses**	1.02	0.15	**bc**	8.47	0.25	**b**	3.64	0.25	**b**	3.34	0.20	**ab**	-26.6	0.1	**bc**	22 261	10 054	a
**Bare sand**	1.53	0.16	**a**	1.56	0.07	**d**	0.50	0.09	**d**	0.42	0.08	**c**	-24.1	0.1	**a**	n.d.	n.d.	
**DUVANNY YAR**																		
**Vegetated Holocene cover**	0.06	0.01	**c**	79.89	6.23	**a**	39.57	1.64	**a**	39.91	2.99	**a**	-29.4	0.6	**b**	n.d.	n.d.	
**Bare freshly thawed Yedoma**	1.68	0.11	**a**	3.85	0.34	**b**	1.04	0.12	**c**	1.03	0.11	**b**	-26.1	1.5	**ab**	n.d.	n.d.	
**Bare earlier thawed Yedoma**	1.17	0.21	**b**	5.20	1.47	**b**	1.97	0.98	**b**	1.65	0.56	**b**	-26.2	0.4	**a**	n.d.	n.d.	
**Yedoma revegetated with mosses**	1.15	0.38	**ab**	6.77	3.03	**b**	2.96	2.57	**bc**	2.27	1.30	**b**	-26.5	0.8	**a**	n.d.	n.d.	
**Yedoma revegetated with grasses**	1.02	0.33	**b**	4.83	1.02	**b**	1.96	0.87	**b**	1.85	0.95	**b**	-27.1	0.9	**a**	n.d.	n.d.	

Supplementary Table 2. – 2/2	Total N content %			$\delta^{15}N_{bulk}$ ‰			C/N ratio			NH_4^+ content mg N (kg DW)$^{-1}$			NO_3^- content mg N (kg DW)$^{-1}$			pH		
	**Mean**	**SD**	**Sig.**	**Mean**	**SD**	**Sig.**	**Mean**	**SD**	**Sig.**	**Mean**	**SD**	**Sig.**	**Mean**	**SD**	**Sig.**	**Mean**	**SD**	**Sig.**
**KURUNGNAKH**																		
**Vegetated Holocene cover**	0.29	0.03	**ac**	1.24	0.25	**a**	38.0	3.7	**a**	8.99	11.73	**bc**	0.00	0.00	**b**	5.7	0.3	**c**
**Bare freshly thawed Yedoma**	0.16	0.03	**acd**	1.35	0.33	**a**	13.7	4.2	**ab**	35.33	8.19	**a**	0.00	0.01	**b**	8.1	0.2	**ab**
**Bare earlier thawed Yedoma**	0.14	0.02	**bd**	0.89	0.17	**a**	14.3	1.7	**ab**	18.43	3.51	**abc**	0.00	0.00	**b**	7.3	0.6	**abc**
**Yedoma revegetated with mosses**	0.14	0.02	**bd**	0.98	0.29	**a**	14.6	1.6	**ab**	27.88	10.79	**ab**	1.10	0.61	**a**	8.3	0.1	**a**
**Yedoma revegetated with grasses**	0.24	0.03	**ab**	1.02	0.13	**a**	14.1	1.1	**ab**	2.60	0.76	**c**	0.69	0.25	**a**	8.2	0.3	**a**
**Bare sand**	0.04	0.01	**d**	0.47	1.12	**a**	10.0	3.3	**b**	15.43	0.77	**abc**	0.00	0.00	**b**	6.6	0.2	**bc**
**DUVANNY YAR**																		
**Vegetated Holocene cover**	0.76	0.13	**a**	0.78	0.87	**b**	54.3	12.0	**a**	3.53	1.15		1.00	2.24		4.6	0.2	**b**
**Bare freshly thawed Yedoma**	0.08	0.01	**c**	3.89	0.64	**a**	12.4	0.8	**ab**	0.93	0.35		0.08	0.10		6.8	0.2	**b**
**Bare earlier thawed Yedoma**	0.15	0.06	**b**	3.01	1.50	**a**	11.3	1.2	**b**	8.51	12.28		39.44	44.05		8.1	0.4	**a**
**Yedoma revegetated with mosses**	0.17	0.06	**ab**	3.33	1.06	**a**	12.8	3.0	**b**	1.88	0.83		7.58	9.57		8.0	0.5	**a**
**Yedoma revegetated with grasses**	0.13	0.03	**bc**	1.27	1.09	**b**	14.2	3.8	**b**	1.34	0.88		0.06	0.12		8.1	0.2	**a**

Supplementary Table 3. Nitrogen (N) transformation rates at the Kurungnakh Yedoma exposure. Samples were taken from the depth of 0–10 cm. Lower case letters indicate statistically significant differences between surfaces (Kruskal-Wallis test with pairwise comparisons with Dunn's test; $p < 0.05$, $n = 5$). DW = dry weight; SD – Standard deviation; n.d. – not determined.

	Gross N mineralization mg N (kg DW)$^{-1}$ h^{-1}			Net N mineralization mg N (kg DW)$^{-1}$ h^{-1}			Net ammonification mg N (kg DW)$^{-1}$ h^{-1}			Net nitrification mg N (kg DW)$^{-1}$ h^{-1}		
	**Mean**	**SD**	**Sig.**	**Mean**	**SD**	**Sig.**	**Mean**	**SD**	**Sig.**	**Mean**	**SD**	**Sig.**
**KURUNGNAKH**												
**Vegetated Holocene cover**	n.d.			–0.87	0.69	**c**	–0.62	0.57	**c**	–0.25	0.12	**c**
**Bare freshly thawed Yedoma**	0.29	0.20	**ab**	–0.15	0.13	**bc**	–0.11	0.13	**bc**	–0.04	0.02	**bc**
**Bare earlier thawed Yedoma**	0.43	0.17	**a**	0.08	0.15	**ab**	0.12	0.14	**ab**	–0.04	0.01	**bs**
**Yedoma revegetated with**	0.13	0.06	**ab**	0.12	0.16	**ab**	0.03	0.14	**ab**	0.08	0.03	**ab**
**Yedoma revegetated with**	0.63	0.42	**a**	0.31	0.06	**a**	0.20	0.05	**a**	0.11	0.01	**a**
**Bare sand**	0.04	0.02	**b**	–0.14	0.03	**bc**	–0.11	0.03	**bc**	–0.03	0.01	**abc**

Supplementary Table 4. Nitrous oxide (N_2O) production rates at the Kurungnakh Yedoma exposure under different headspace conditions. Samples were taken from the depth of 0–10 cm and incubated under field moisture content at 10 °C. Lower case letters indicate statistically significant differences between surfaces (Kruskal-Wallis test with pairwise comparisons with Dunn's test; $p < 0.05$, $n = 5$). DW = dry weight; SD – standard deviation; n.d. – not determined.

	N_2O production ng N (kg DW)$^{-1}$ h^{-1}								
**Headspace treatment**	**Oxic**			**Anoxic**			**Anoxic with acetylene**		
	**Mean**	**SD**	**Sig.**	**Mean**	**SD**	**Sig.**	**Mean**	**SD**	**Sig.**
**KURUNGNAKH**									
**Vegetated Holocene cover**	0.005	0.002	**a**	0.26	0.10	**bc**	0.43	0.09	**bc**
**Bare freshly thawed Yedoma**	0.001	0.006	**ab**	0.80	0.15	**ab**	0.25	0.22	**c**
**Bare earlier thawed Yedoma**	–0.008	0.009	**b**	0.13	0.02	**c**	0.42	0.04	**bc**
**Yedoma revegetated with mosses**	0.005	0.004	**ab**	3.97	4.83	**ab**	16.40	23.72	**ab**
**Yedoma revegetated with grasses**	0.006	0.004	**a**	25.27	17.83	**a**	117.54	78.98	**a**
**Bare sand**	n.d.			n.d.			n.d.		

Supplementary Table 5. Primer pairs used in this study and corresponding amplification protocols.

Specificity	Primer	Sequence (5'-3')	Thermal conditions	Ref.
Archaeal *amoA*	Cren-amoA-19F	ATGGTCTGGYTWAGACG	(95°C, 1 min) x 1, (95°C, 10s; 56°C, 20s; 72°C, 15s) x 40	1
	TamoA-632R-4	GCKGCCATCCATCKRTANGTCCA		2
Bacterial *amoA*	1F	GGGGTTTCTACTGGTGGT	(95°C, 1 min) x 1, (95°C, 10s;60°C, 20s; 72°C, 15s) x 40	3
	2R	CCCCTCKGSAAAGCCTTCTTC		
16S *rRNA*	338 F	CCTACGGGAGGCAGCAG	(95°C, 1 min) x 1, (95°C, 10s; 60°C, 20s; 72°C, 15s) x 40	4
	518 R	ATTACCGCGGCTGCTGG		

[1] Modified from Leininger et al (2006)
[2] Siljanen et al. (2019)
[3] Rotthauwe et al. (1997)
[4] Muyzer et al. (1993)

Supplementary Notes

Supplementary Note 1. Extended methods.

Study sites

The Kurungnakh exposure (N 72°20', E 126°17') is located on the Eastern shore of Kurungnakh-Sise Island in the Lena River Delta, Northeast Russia (Fig, 1; Supplementary Fig. S1). The area is underlain by continuous permafrost [1], and the climate is continental Arctic. The mean annual air temperature at the nearby research station Samoylov Island (N 72°22', E 126°29') is –12.3 °C and annual rainfall amounts to 169 mm [2]. Kurungnakh Island is elevated up to 55 m a.s.l. [1], and mostly composed of ice-rich Yedoma covering fluvial sandy deposits [3]. Detailed information about the climatic conditions in the region [2] and depositional characteristics of Kurungnakh Island [3] can be found in the literature. Altogether, 30 plots representing six different surface types in five replicates were chosen for the study on Kurungnakh. The studied surface types were: 1) Holocene cover deposits overlying undisturbed Yedoma permafrost on the top of the exposure, densely vegetated with moss (*Hylocomium obtusifolium*), sedge (*Carex concolor*) and dwarf shrub (*Salix pulchra*)

communities; 2) freshly thawed Yedoma bare of vegetation, close to thawing ice-wedges in the upper part of the exposure; 3) earlier thawed Yedoma bare of vegetation; 4) disturbed Yedoma in the lower, stabilized parts of the slope, revegetated by mosses *Funaria hygrometrica* and *Ceratodon purpureus*; 5) disturbed Yedoma revegetated by grass (*Arctagrostis arundinacea*); and 6) bare sand close to the river shore receiving Yedoma melt-waters by a small stream running through the exposure (Supplementary Fig. S1). In addition to above-mentioned mosses and grasses, herbs *Descurainia sophioides* and *Tephroseris palustris*, both typical pioneering plants for Yedoma exposures, were adundant at the Kurungnakh exposure**.**

The Duvanny Yar exposure (68°38' N, 159°09' E) is located on the right bank of the Kolyma River, Northeast Russia (Fig. 1; Supplementary Fig. S2). The area is underlain by continuous permafrost, and the climate is continental Arctic. The mean annual air temperature in the region is −11 °C and annual rainfall is 197 mm [4]. Detailed information of the depositional characteristics at the site can be found in the literature [5, 6]. In Duvanny Yar, 35 plots representing seven different surface types in five replicates were studied: 1) Holocene cover deposits overlying undisturbed Yedoma permafrost on the top of the exposure, where open larch forest is composed of *Larix dahurica* in tree layer with dwarf shrubs (*Ledum palustre*, *Betula exilis*., *Salix* sp., *Vaccinium* spp.*)*, mosses and lichens in the understory [7]; 2) wet, freshly thawed Yedoma bare of vegetation, close to thawing ice-wedges in the upper part of the exposure; 3) dry, earlier thawed Yedoma bare of vegetation; 4) moist, earlier thawed Yedoma bare of vegetation; 5) disturbed and revegetated Yedoma in the lower, stabilized parts of the slope, colonized by mosses *Funaria hygrometrica* and *Ceratodon purpureus*; 6) and 7) disturbed Yedoma revegetated by lower and taller canopies, respectively, of grasses *Arctagrostis arundinacea* and *Puccinella neglecta* (Supplementary Fig. S2). For the data analysis, the two bare earlier thawed surfaces 3) and 4) as well as the two revegetated surfaces with grasses 6) and 7) were merged in order to match with the Kurungnakh data.

In situ N_2O fluxes

In situ nitrous oxide (N_2O) fluxes were measured by the static chamber technique [8], twice in July 2016 on Kurungnakh and once in July 2017 in Duvanny Yar. Although N_2O emissions are known to commonly occur as high pulses or hot moments often outside the peak summer period [9, 10], our own studies on bare peat surfaces have shown that temporal pattern of the

emissions in these hot spots follows closely the temperature variations, peaking at high summer [11]. This (besides obvious logistical reasons) guided our selection of timing for the field campaign since we wanted to catch the peak emissions and thus maximize the emission range to reveal the drivers of spatial variability. The two measurement rounds in Kurungnakh confirm that the spatial patterns observed in N_2O emissions were robust with respect to timing: the correlation between the two measurement occasions was very high (Pearson correlation with log-transformed data, $R = 0.92$, $p < 0.001$). Another challenge related to soil N_2O measurements is the large spatial variability [10]. Plot selection for manual chamber studies requires usually compromising between the number of studied surface types and the replicate number; here, we used the replication of five, which has proved sufficient in our previous studies [12].

Aluminum chambers with a headspace volume of 29 dm^3 and a base area of 0.10 m^2 were pushed into the soil down to a depth of 2–5 cm for the time of the measurement. The chamber headspace was continuously mixed with a fan, and a coiled outlet tube (i.d. 2 mm, length 1.5 m) was equilibrating the pressure difference between the atmosphere and the chamber headspace. Five gas samples were drawn from the chamber headspace within a 50-minute enclosure time. The gas samples were transferred with a needle through a rubber septum into pre-evacuated 12 ml glass vials (Labco), sealed with hot-melt glue, and shipped to the laboratory of the University of Eastern Finland for gas analysis, which was conducted ~6 months after the sampling. The storage method used here has been earlier proven suitable for storage time of several months [13]. At the time of gas sampling, the following auxiliary measurements were conducted: chamber height, chamber temperature (thermometer TM-80N with a K-type thermocouple probe, Tenmars, Taipei), and soil moisture as volumetric water content (VWC) in the topsoil (0–7 cm; ML3 ThetaProbe, Delta-T Devices, Cambridge, UK).

The gas mixing ratios of the samples were determined with a gas chromatograph (GC; Agilent 7890B Agilent Technologies, Santa Clara, CA, USA) equipped with an autosampler (Gilson Inc., WI, Middleton, USA), an electron capture detector (ECD) for N_2O and a flame ionization detector (FID) for methane (CH_4). The mixing ratios (ppm) were calculated from the peak areas based on a standard curve with 5 different levels of N_2O concentrations, ranging from 0 to 5000 ppb. Fluxes of N_2O were calculated from the slope of the linear increase of the

N_2O mixing ratio in the chamber headspace as a function of time. Clearly erroneous sampling points were occasionally discarded from the regression. The quality control of gas flux results was based on inspection of Root Mean Square Error (RMSE) in ppm as compared to the variability of standard gas mixtures in a similar range. The fluxes with RMSE > 3 * SD of the standard were discarded.

Flux of carbon dioxide (CO_2) in the dark, representing ecosystem respiration (ER) that includes both soil respiration by soil microbes and autotrophic respiration by plant roots and shoots, was measured with the dynamic chamber technique [12]. The same chambers as for the N_2O measurements were used. The headspace gas was continuously circulated through an infrared gas analyzer (Li-840, LiCor Lincoln, Nebraska, USA on Kurungnakh; EGM-4, PP Systems, Amesbury, MA, USA in Duvanny Yar) which determined and logged the CO_2 concentrations at 1 (in 2016) to 5 (in 2017) s intervals. The rate of CO_2 flux was determined from the phase of linear increase within the 2min measurement time. Non-linear concentration data in the beginning of the sampling, caused by disturbance of pushing the chamber into the ground, were discarded.

Soil pore gas sampling and N_2O flux estimation with a regression function

Soil pore gas at 10 cm depth was sampled at the revegetated Yedoma surfaces with a stainless-steel probe equipped with a three-way stopcock to allow tight connection with the sampling syringe. Water saturated conditions or high bulk density of the soil prevented soil pore gas sampling at most of the bare Yedoma surfaces as well as bare sand. The gas samples were stored and analyzed as described above. We used the dependence between soil pore N_2O concentration at the depth of 10 cm and N_2O flux observed at revegetated Yedoma surfaces of the two study sites (Supplementary Figure S5). Both parameters were log-transformed before fitting a linear regression to the data. The regression was able to explain 80% of the variability in the N_2O fluxes from revegetated sites.

Soil sampling and analysis

After the chamber measurements, soil samples were taken from the topsoil (0–10 cm) of each chamber plot using a steel corer (i.d. 8.5 cm). Roots and other plant parts and stones were removed, after which the soils were homogenized by sieving (mineral soils; 5 mm mesh size) or by hand-mixing (organic soils).

Bulk density was determined from volumetric soil samples after drying until constant weight at 60 or 105 °C for organic and mineral soils, respectively. The dried soil was used in determination of particle density and content of soil organic matter (SOM), carbon (C) and nitrogen (N). Particle density was determined from dry, finely ground soil by a pycnometric method. For elemental analysis, dried soil was homogenized in a ball mill (Retsch MM301, Haan, Germany). Total content of C, organic C and N, as well as $\delta^{13}C$ of SOC and $\delta^{15}N$ in the bulk soil were analyzed with an elemental analyzer (Thermo Finnigan Flash EA 1112 Series, San Jose, CA, USA). For organic C analysis, inorganic C was removed from a subsample with the acid fumigation method [14]. Water-filled pore space (WFPS) was calculated from VWC measured *in situ*, using bulk density and particle density determined as described above. Soil pH was measured from slurries with a soil:H_2O ratio of 1:4 ratio (w/v).

For determination of mineral N content, ammonium (NH_4^+) nitrate (NO_3^-) were extracted from freshly sampled soils at the field laboratory with 1 M KCl using a 1:3 volume ratio of soil to KCl solution. After 1 h rotatory shaking at 175 rpm, the extracts were filtered with Whatman 589 filterpapers. The extracts were frozen for storage and shipment to the laboratory of the University of Eastern Finland, where the mineral N concentrations in the extracts were determined by spectrophotometric methods as previously described [12]. The determination of NO_3^- concentration is based on the Griess reaction [15], the determination of NH_4^+ concentration is based on sodium phenate-hypochlorite reaction [16]. Sample absorbance at 540 nm and 650 nm wavelengths for NO_3^- and NH_4^+, respectively, were measured with a microplate reader (Wallac, 1420 VICTOR3, Perkin Elmer, Turku, Finland).

Gross and net N transformation rates

Nitrogen transformation rates were determined in the field laboratory from freshly sampled soils. For determination of gross N mineralization and nitrification rates, we used the pool dilution method, which is based on labelling the product pool (NH_4^+ for mineralization, NO_3^- for nitrification) with the heavy N isotope, ^{15}N, and following the dilution in the isotopic label with time [17, 18]. However, due to low mineral N content and high N immobilization of the studied soils, we were not able to detect the dilution of the ^{15}N stable isotope as a result of gross nitrification in any of the studied soils, and also determination of gross mineralization failed in some soil types or replicates. However, we could use the same experiment to calculate the net N mineralization and nitrification rates as described below. The net rates

represent the net balance of consumption and production of inorganic N species and, thus, describe well the N saturation of the system. The initial N addition amounted to 2.1–2.6 mg N (kg DW)$^{-1}$ depending on the soil moisture content. Since the N addition was targeted to the product, not the precursor pool of each net N transformation process, we have good reasons to believe it did not significantly affect the net transformation rates. However, it may have led to more negative net mineralization and nitrification rates in the soils showing net N immobilization, such as vegetated Holocene cover and freshly thawed Yedoma.

Two sets of samples were prepared for both N mineralization and nitrification measurements by weighing 2 g fresh weight of homogenized soil into plastic tubes with screw caps. 500 µl of 0.25 mM, 10 at-% $(^{15}NH_4)_2SO_4$ solution was added to the N mineralization samples, whereas for nitrification samples, 500 µl of 0.50 mM, 10 at-% $K^{15}NO_3$ solution was used. After labelling, the samples were incubated for 24 h at the approximate *in situ* temperature of ~5 °C. Nutrient levels (NO_3^- and NH_4^+) were determined from samples extracted at two time points of 4 and 24 hours with 2 M KCl. The extracts were frozen to –18°C for the transport and kept frozen until the analysis. Concentrations of NH_4^+ and NO_3^- in the extracts were determined spectrophotometrically as described above. Content of ^{15}N content in NH_4^+ extracts was analyzed by continuous-flow isotope ratio mass spectrometer (IRMS; Thermo Finnigan DELTA XPPlus, San Jose, CA, USA) coupled to an elemental analyzer (Thermo Finnigan Flash EA 1112 Series) and an open split interface (Thermo Finnigan Conflow III) after conversion to solid phase by the microdiffusion method as previously described [11].

Gross N mineralization rates per mass of dry soil were calculated based on a pool dilution model [17-19]. Net N mineralization rates were calculated by dividing the difference of the total mineral N content (NH_4^+ and NO_3^-) between the first and second sampling points with the incubation time. Net ammonification and nitrification rates were calculated similarly from the change in NH_4^+ and NO_3^- contents, respectively.

N_2O production and total denitrification rates in laboratory incubations

Experiment 1: Nitrous oxide production under different headspace conditions

Rates of N_2O production and total denitrification were determined from Kurungnakh soil samples, which had been kept frozen during storage and shipment to the University of Eastern Finland. After thawing the soils, the soils were pre-incubated at 4 °C before homogenization by hand mixing and removal of visible roots. For the incubation experiments, 10 g fresh weight

of organic soil (Holocene cover) and 25 g fresh weight (FW) of mineral soils (bare and revegetated Yedoma and sand) were weighed into 120 ml glass flasks. The flasks were closed with parafilm with small holes to allow gas exchange but prevent loss of moisture from soil and kept at 10 ^{o}C for three days to allow the soils to acclimatize to the incubation temperature.

The soils were incubated at field moisture content at 10 ^{o}C for six days in five replicates under three different headspace treatments: 1) oxic, 2) anoxic and 3) anoxic with acetylene. For oxic treatment (1), the flasks were flushed with laboratory air before closing them with screw-caps equipped with thick butyl rubber septa (GL45, Glasgerätebau OCHS) to allow gas sampling during the incubation. For anoxic treatments with and without acetylene (2 and 3), the flasks were closed inside a glove bag after flushing several times with N_2 gas (purity ≥ 99.999%). The rubber septa used for these anoxic treatments had been depleted from oxygen by keeping them in the glove bag in the N_2 headspace overnight. In addition to sample flasks, 3 blank samples without soil were added per each treatment. To confirm the anoxic conditions in the anoxic treatments, color indicator strips (Microbiologic Anaerotest, Merck) were placed inside the flasks. Acetylene was added into flask headspace in the third treatment at 10 vol-% in order to block the last step of denitrification, reduction of N_2O to N_2, thus making N_2O as the final denitrification product [20]. Anoxic flasks were kept in a water bath to minimize the risk of oxygen exposure.

Gas samples were taken at five time points at days 0, 1, 2, 3 and 6 after the start of the incubation. To allow repeated sampling of 25 ml gas samples, we overpressurized the flasks before each sampling with a corresponding amount of headspace gas. Concentrations of N_2O in the gas samples were determined with GC as described above. Due to intense accumulation of N_2O for some soil types and treatments, a linearity test was performed to confirm the linearity of the GC-ECD up to 2000 ppm N_2O (data not shown). We calculated the rate of N_2O production per mass of dry soil for each sampling interval.

For oxic treatment, flux was calculated from the slope of a linear regression fitted to the first four sampling points. Within this time range, the N_2O production rate was constant, after which it evened out in many of the samples. For the anoxic treatments, we report the maximum N_2O production between two sampling points, because we often observed N_2O

consumption (treatment 2 without acetylene) or steady state (treatment 3 with acetylene) after initial N_2O production, indicating reduction of N_2O to N_2.

Experiment 2: Response of nitrous oxide production to different moisture conditions and carbon and nitrogen sources

The aim of the second incubation experiment was to investigate the factors limiting N_2O production in freshly thawed Yedoma. We dried the freshly thawed Yedoma to reduce the water content by 25%, weighed 17 g FW to incubation flasks, and incubated under three different headspace treatments as described above. For each headspace treatment, we applied three different amendments within a volume of 250 µl per flask: control (milli-Q H_2O addition), addition of C (glucose; 67 µg C (g DW)$^{-1}$, equal to 0.3% of SOC), or addition of C (as above) and NO_3^- (4.7 µg N (g DW)$^{-1}$, equal to 0.3% of TN). The GC analysis and calculation followed the procedure described above for Experiment 1.

Molecular studies on microbial community participating in N cycling

Nucleic acid extraction and purification

For molecular studies, we used three biological replicates from five surface types on Kurungnakh, representing different stages of thermokarst and post-thaw succession: vegetated Holocene cover, freshly or earlier thawed Yedoma, revegetated Yedoma with mosses or with grasses. We extracted DNA from these samples in three technical replicates as described previously [21]. In brief, DNA samples were extracted with phenol/chloroform/IAA extraction followed by NaCl/PEG3000 precipitation and purification with PVP/Sephadex/Sepharose columns. The lab replicates were then pooled by surface prior to purification and stored in deep freeze at –80 °C until the analysis.

Quantification of amoA and 16S rRNA genes

Quantitative PCR (qPCR) of archaeal and bacterial *amoA* and 16S *rRNA* genes was performed using the reaction and cycling conditions as described previously and summarized in Supplementary Table S6 [21-24]. All reactions were performed in duplicates. Quantification of archaeal and bacterial *amoA* genes was based on 10-fold dilutions (10^1–10^8) of *amoA* gene fragments from *N. viennensis* EN76 and *N. multiformis*, respectively, amplified from cloned gene fragment with vector specific primers. For archaeal *amoA* genes, the qPCR efficiency was 75.5%, with a detection limit between $6.55*10^2$ and $6.55*10^7$ genes per reaction (25 µl). For bacterial *amoA* genes, the efficiency was 91.1%, with a detection limit between $1.88*10^3$ and

$1.88*10^8$ genes per reaction. For 16S rRNA the efficiency was 83.1% with a detection limit between $2.4*10^4$ and $2.5*10^9$ genes per reaction. The specificity of qPCR amplification products was verified by melting-curve analysis and gel electrophoresis.

Captured metagenomics of N cycling microbes

To detect the changes in N-cycling-relevant microbial community structure with permafrost thaw and post-thaw succession, we studied the relative abundances of the key N cycling genes using a captured metagenomic tool. The method has been validated and tested for overall performance and specificity of the probes used for sequence capture [25], and it has been successfully applied for studying N cycling genes in bioreactors treating aquaculture effluents [26]. This method is designed for targeting and sequencing the organisms carrying the key N cycling genes involved in the following processes: N_2 fixation (*nifH*), nitrification (*amoA*), NO_3^- reduction (*narG, napA*), denitrification (*nirK, nirS, norB, nosZ*), dissimilatory nitrate reduction to ammonium (DNRA) (*nrfA*) and anammox (*hdhA*) using gene specific probes following the NimbleGen SeqCap EZ protocol by Roche NimbleGen, Inc.

To develop sequence capturing tool, all known sequences for the targeted nitrogen cycling genes were collected from publicly available (NCBI, WGS, Fungene) and private databases with gene specific probes into a local server (CSC, Espoo, Finland). These functional genes were then searched for selected areas of genes with nhmmer [27] and tblastx [27, 28] with manually curated and aligned databases of known and isolated clades of each gene. These target databases (TDBs) were then submitted into SeqCap pipeline with default parameters for designing up to six unique 50 mer probes for each cluster (with 90% cut-off) of each gene. Information of these probes were then used to design NimbleGen SeqCap probe set (approximately 2M probes) for Captured metagenomics. Performance and specificity of these probes for sequence capture was validated and tested [25]. For each sample, libraries with Indexes and sequencing adapters were produced in CGR (Liverpool Centre for Genomic Research, Liverpool, UK). These libraries were then pooled together uniformly, quality controlled and hybridized to the NimbleGen SeqCap capturing probe set according manufacturer's instructions at 47°C for 72 hours in CGR as previously described [25]. The sequencing produced about 2 million reads per sample. To diminish the computing effort the sequence files were split according to each functional gene by using the target gene hmmer profiles to search sequences of each functional gene. Maximum E-value cut-off ($E < 0.001$)

was used. The gene functions and closest cultured relatives of each sequence were searched against Swiss-prot database with tblastx algorithm in diamond sequence search accelerator [29].

Statistical analysis

All statistics were conducted with R, version 3.6.1 [30]. We tested normal distribution of N_2O fluxes, soil physico-chemical characteristics and N transformation rates by visual inspection of histograms and Q-Q plots and with the Shapiro-Wilk normality test of the *stats* package [31]. Many of the parameters were not normally distributed even after transformation (logarithmic, square root), so we chose the non-parametric Kruskall-Wallis test [31] for testing the differences between studied surfaces, followed by pairwise comparisons with Dunn's test of the *FSA* package [32]. For normally distributed data, we tested the equality of variances with the Bartlett's test [31]. If the variances were equal, we tested the group differences with the Welch's one-way analysis of variance (ANOVA) [31] followed by pair-wise comparisons with the Games-Howell post-hoc test of the *userfriendlyscience* package [33]. The group differences between the studied surface types were tested separately within each study site (fluxes, soil characteristics) or for each treatment (N_2O production in soil incubations). The Kruskall-Wallis test with Dunn's post hoc tests was also used to test differences in molecular data (relative abundances, copy numbers & gene ratios; n = 3) between the studied soils. Treatment differences in the incubation experiments were tested with Wilcoxon signed rank test. The role of soil characteristics, mineral N content and N transformation rates as drivers of *in situ* N_2O fluxes was explored by the non-parametric Spearman correlation analysis with the *Hmisc* package [34].

Rate and volume of thermal erosion and related N mobilization at the Kurungnakh exposure

We calculated the rate and extent of thermal erosion for the south-eastern part of the riverbank on Kurungnakh-Sise Island (length of the section 1.7 km) in QGIS v 3.14 (with SAGA and GRASS) via the following steps:

First, we delineated the shoreline in 2019 based on a digital elevation model (DEM) from unmanned aerial vehicle (UAV) imaging from July 2019. We automatically built a contour at a

certain height on the river shore and then approximated the shoreline by setting points along this contour at equal distances (0.5 m).

Second, we determined the Yedoma cliff boundary on the top of the exposure based on the DEM and orthophoto mosaic derived from UAV imaging conducted in summer 2019. On the cliff boundary, we placed points at equal distances (5 m). We removed points that were in erosional gullies so that they are excluded from calculations.

Third, we calculated distances (straight lines) from each point on the cliff in 2019 to the nearest point on the shoreline and built intersection points for these lines with the cliff boundary determined from Arctic DEM datasets from March 2012 and April 2014. This ArcticDEM strip dataset was based on stereophotogrammetric DEM processing [35]. We split lines from the previous step with these intersection points and removed line sections that connect 2012-2014 cliff boundary with the shoreline. The length of the remaining lines represents the Yedoma retreat between 2012-2014 and 2014-2019. This algorithm resembles procedures that are available from the free AMBUR package [36]. The resulting data showed that between 2012 and 2019 the Yedoma boundary retreated as a result of thermal erosion by 1.4–70.3 m depending on the location, with a median (with 25th–75th percentiles) of 3.7 (2.5–5.7) m year^{-1}.

Finally, we shifted Arctic DEMs from 2012 and 2014 to match UAV DEM from 2019, both horizontally and vertically. To do this we used stable baydzherakhs in DEMs as reference points (this is convenient because in winter baydzherakh tops seldom have snow cover due to wind blowing it off). Then we calculated the volume of eroded material along the studied cliff boundary from DEM difference (DEM-2019 subtracted from ArcticDEM-2014 and ArcticDEM-2012; the resulting value, if positive, means subsidence due to thaw and erosion). We did the calculation in QGIS, using the 'Raster surface volume instrument'. The resulting volume estimate, 223 502 m^3 (89 113 m^3 between 2012 and 2014; 134 389 m^3 between 2014 and 2019), is a conservative one because the calculation was in the area between the two cliff boundaries and does not include the thermal erosion in the lower part of the slope. Despite the fact that ArcticDEM data from March 2012 and April 2014 contains snow, there were no pronounced zones of snow accumulation in the area to which we limited the calculation.

We further approximated how much N was annually liberated at the Kurungnakh exposure as a result of permafrost thaw (m_N; kg m^{-1} $year^{-1}$). First, we calculated the annually eroded volume per unit area of the cliff based on the total eroded volume ($V_{degraded}$ = 223 502 m^3), the width of the cliff (width = 1720 m) and the mean rate of cliff retreat (rate = 3.7 m $year^{-1}$). We used the total ground-ice content (*IC* = 82 vol %) [37], and assumed a negligible N content in the ground-ice. We multiplied the remaining sediment volume (18 vol %) with the bulk density (*BD;* 1.22 g cm^{-3}) and total N content (*%N*; 0.16 %) detected in freshly thawed Yedoma in this study (Supplementary Table S2):

$$m_N = (1 - IC/100) * V_{degraded} \text{ / width / rate } * BD * \%N \qquad (1)$$

This resulted in N release from thawing permafrost at the Kurungnakh cliff boundary of 1.7 kg m^{-2} $year^{-1}$. Using similar approach, we estimated the direct release of mineral N at permafrost thaw using the NH_4^+ content in freshly thawed yedoma of 35.3 mg N (kg DW)$^{-1}$ (Supplementary Table S2).

References

1. Morgenstern, A., Grosse, G., Guenther, F., Fedorova, I. & Schirrmeister, L. Spatial analyses of thermokarst lakes and basins in Yedoma landscapes of the Lena Delta. *Cryosphere* **5**, 849-867 (2011).

2. Boike, J. *et al*. A 16-year record (2002-2017) of permafrost, active-layer, and meteorological conditions at the Samoylov Island Arctic permafrost research site, Lena River delta, northern Siberia: an opportunity to validate remote-sensing data and land surface, snow, and permafrost models. *Earth Syst. Sci. Data* **11**, 261-299 (2019).

3. Wetterich, S. *et al*. Palaeoenvironmental dynamics inferred from late Quaternary permafrost deposits on Kurungnakh Island, Lena Delta, Northeast Siberia, Russia. *Quat. Sci. Rev.* **27**, 1523-1540 (2008).

4. Göckede, M. *et al*. Shifted energy fluxes, increased Bowen ratios, and reduced thaw depths linked with drainage-induced changes in permafrost ecosystem structure. *Cryosphere* **11**, 2975–2996 (2017).

5. Strauss, J., Schirrmeister, L., Wetterich, S., Borchers, A. & Davydov, S. P. Grain-size properties and organic-carbon stock of Yedoma Ice Complex permafrost from the Kolyma lowland, northeastern Siberia. *Global Biogeochem. Cycles* **26**, GB004104 (2012).

6. Murton, J. B. *et al*. Palaeoenvironmental Interpretation of Yedoma Silt (Ice Complex) Deposition as Cold-Climate Loess, Duvanny Yar, Northeast Siberia. *Permafr. Periglac. Process.* **26**, 208-288 (2015).

7. Smith, C. A. S. *et al*. A description and classification of soils and landscapes of the lower Kolyma River, Northeastern Russia. *Polar Geogr.* **19**, 107-126 (2015).

8. Marushchak, M. E. *et al*. Hot spots for nitrous oxide emissions found in different types of permafrost peatlands. *Glob. Chang. Biol.* **17**, 2601-2614 (2011).

9. Wagner-Riddle, C. *et al*. Globally important nitrous oxide emissions from croplands induced by freeze–thaw cycles. *Nature geoscience* **10**, 279-283 (2017).

10. Butterbach-Bahl, K., Baggs, E. M., Dannenmann, M., Kiese, R. & Zechmeister-Boltenstern, S. Nitrous oxide emissions from soils: how well do we understand the processes and their controls? *Philos. Trans. R. Soc. B-Biol. Sci.* **368** (2013).

11. Voigt, C. *et al*. Nitrous oxide emissions from permafrost-affected soils. *Nat. Rev. Earth Environ* **1**, 420–434 (2020).

12. Voigt, C. *et al*. Warming of subarctic tundra increases emissions of all three important greenhouse gases - carbon dioxide, methane, and nitrous oxide. *Glob. Chang. Biol.* **23**, 3121-3138 (2017).

13. Repo, M. E. *et al*. Large N_2O emissions from cryoturbated peat soil in tundra. *Nat. Geosci.* **2**, 189-192 (2009).

14. Ramnarine, R., Voroney, R. P., Wagner-Riddle, C. & Dunfield, K. E. Carbonate removal by acid fumigation for measuring the $\delta^{13}C$ of soil organic carbon. *Can. J. Soil Sci.* **91**, 247-250 (2011).

15. Miranda, K. M., Espey, M. G. & Wink, D. A. A rapid, simple spectrophotometric method for simultaneous detection of nitrate and nitrite. *Nitric Oxide* **5**, 62-71 (2001).

16. Fawcett, J. K. & Scott, J. E. A rapid and precise method for the determination of urea. *J. Clin. Pathol.* **13**, 156-159 (1960).

17. Kirkham, D. & Bartholomew, W. V. Equations for Following Nutrient Transformations in Soil, Utilizing Tracer Data I. *Soil Sci. Soc. Am. J.* **18**, 33 (1954).

18. Kirkham, D. & Bartholomew, W. V. Equations for Following Nutrient Transformations in Soil, Utilizing Tracer Data: II. *Soil Sci. Soc. Am. J.* **18**, 33 (1955).

19. Mary, B., Recous, S. & Robin, D. A model for calculating nitrogen fluxes in soil using ^{15}N tracing. *Soil Biol. Biochem.* **30**, 1963-1979 (1998).

20. Klemedtsson, L., Svensson, B. H. & Rosswall, T. A Method of Selective-Inhibition to Distinguish between Nitrification and Denitrification as Sources of Nitrous-Oxide in Soil. *Biol. Fertil. Soils* **6**, 112-119 (1988).

21. Siljanen, H. M. P. *et al*. Archaeal nitrification is a key driver of high nitrous oxide emissions from arctic peatlands. *Soil Biology & Biochemistry* **137**, UNSP 107539 (2019).

22. Leininger, S. *et al*. Archaea predominate among ammonia-oxidizing prokaryotes in soils. *Nature* **442**, 806-809 (2006).

23. Rotthauwe, J. H., Witzel, K. P. & Liesack, W. The ammonia monooxygenase structural gene amoA as a functional marker: Molecular fine-scale analysis of natural ammonia-oxidizing populations. *Appl. Environ. Microbiol.* **63**, 4704-4712 (1997).

24. Muyzer, G., Dewaal, E. C. & Uitterlinden, A. G. Profiling of Complex Microbial-Populations by Denaturing Gradient Gel-Electrophoresis Analysis of Polymerase Chain Reaction-Amplified Genes-Coding for 16s Ribosomal-Rna. *Appl. Environ. Microbiol.* **59**, 695-700 (1993).

25. Siljanen, H. *et al*. Captured metagenomics on inorganic nitrogen and methane cycling reveals relative abundance and diversity of functional genes. Unpublished; preprint available at URL https://www.dropbox.com/s/uucgyrniwxyb95q/Siljanen_2019b_in-prep_Capture_C-N_metagenomics_MS.pdf?dl=0. (2019).

26. Aalto, S. L. *et al*. Nitrate removal microbiology in woodchip bioreactors: A case-study with full-scale bioreactors treating aquaculture effluents. *Sci. Total Environ.* **723**, 138093 (2020).

27. Wheeler, T. J. & Eddy, S. R. nhmmer: DNA homology search with profile HMMs. *Bioinformatics* **29**, 2487-2489 (2013).

28. Altschul, S. F., Gish, W., Miller, W., Myers, E. W. & Lipman, D. J. Basic Local Alignment Search Tool. *J. Mol. Biol.* **215**, 403-410 (1990).

29. Buchfink, B., Xie, C. & Huson, D. H. Fast and sensitive protein alignment using DIAMOND. *Nature Methods* **12**, 59-60 (2015).

30. R Core Team. R-3.6.1 for Windows (32/64 bit). (2019).

31. R Core Team. The R Stats Package. (2019).

32. Ogle, D., Wheeler, P. & Dinno, A. FSA: Simple Fisheries Stock Assessment Methods. *R package version 0.7.2* (2020).

33. Peters, G., Verboon, P. & Green, J. userfriendlyscience: Quantitative Analysis Made Accessible. *R package version 0.7.2* (2018).

34. Harrell Jr, F. E. Hmisc: Harrell Miscellaneous. *R package version 4.4-1* (2020).

35. Porter, C. *et al*. ArcticDEM. *Harvard Dataverse, V1, January 2021.* (2018).

36. Jackson, C. W., Jr., Alexander, C. R. & Bush, D. M. Application of the AMBUR R package for spatio-temporal analysis of shoreline change: Jekyll Island, Georgia, USA. *Comput. Geosci.* **41**, 199-207 (2012).

37. Strauss, J. *et al*. The deep permafrost carbon pool of the Yedoma region in Siberia and Alaska. *Geophys. Res. Lett.* **40**, 6165-6170 (2013).
